# Comparative Study of Machine-Learning Frameworks for the Elaboration of Feed-Forward Neural Networks by Varying the Complexity of Impedimetric Datasets Synthesized Using Eddy Current Sensors for the Characterization of Bi-Metallic Coins

**DOI:** 10.3390/s22041312

**Published:** 2022-02-09

**Authors:** Rohan Munjal, Sohaib Arif, Frank Wendler, Olfa Kanoun

**Affiliations:** Professorship of Measurement and Sensor Technology, Chemnitz University of Technology, 09126 Chemnitz, Germany; rohan.munjal@etit.tu-chemnitz.de (R.M.); sohaib.arif@s2016.tu-chemnitz.de (S.A.); frank.wendler@etit.tu-chemnitz.de (F.W.)

**Keywords:** eddy current sensor, impedance spectroscopy, machine learning, neural network, comparative study, Keras, Tensorflow, Pytorch, CNTK

## Abstract

A suitable framework for the development of artificial neural networks is important because it decides the level of accuracy, which can be reached for a certain dataset and increases the certainty about the reached classification results. In this paper, we conduct a comparative study for the performance of four frameworks, Keras with TensorFlow, Pytorch, TensorFlow, and Cognitive Toolkit (CNTK), for the elaboration of neural networks. The number of neurons in the hidden layer of the neural networks is varied from 8 to 64 to understand its effect on the performance metrics of the frameworks. A test dataset is synthesized using an analytical model and real measured impedance spectra by an eddy current sensor coil on EUR 2 and TRY 1 coins. The dataset has been extended by using a novel method based on interpolation technique to create datasets with different difficulty levels to replicate the scenario with a good imitation of EUR 2 coins and to investigate the limit of the prediction accuracy. It was observed that the compared frameworks have high accuracy performance for a lower level of difficulty in the dataset. As the difficulty in the dataset is raised, there was a drop in the accuracy of CNTK and Keras with TensorFlow depending upon the number of neurons in the hidden layers. It was observed that CNTK has the overall worst accuracy performance with an increase in the difficulty level of the datasets. Therefore, the major comparison was confined to Pytorch and TensorFlow. It was observed for Pytorch and TensorFlow with 32 and 64 neurons in hidden layers that there is a minor drop in the accuracy with an increase in the difficulty level of the dataset and was above 90% until both the coins were 80% closer to each other in terms of electrical and magnetic properties. However, Pytorch with 32 neurons in the hidden layer has a reduction in model size by 70% and 16.3% and predicts the class, 73.6% and 15.6% faster in comparison to TensorFlow and Pytorch with 64 neurons.

## 1. Introduction

Bi-metallic coins comprise different types of metals within the layered structure, thus embedded with different security features depending upon the fused metal composition and their magnetic properties. To access the magnetic properties from different layers of these coins, multi-frequency excitation signals can be used to excite an eddy current sensor coil in the presence of the coin. Depending upon the used frequencies, a particular penetration depth of the eddy current effect in the coin can be achieved, thus measuring the response in the form of inductance from the varying properties of a coin at different layers. The multi-frequency inductive response of the bi-metallic coins can be further used for their classification into different classes.

For a successful coin classification with a high accuracy rate, decision boundaries are needed to be created in an optimal way, which can be created using machine learning techniques based on artificial neural networks (ANNs). However, for the implementation of neural networks, it is recommended to use a huge dataset for the model training, to achieve better prediction accuracy. Since the availability of bi-metallic coins is limited, therefore, it poses a challenge to measure and create a huge dataset for the learning of a neural network (NN) with a limited number of coins. This problem can be overcome by real measurements of available coins using an eddy current sensor and further use of the measured impedance spectra of the coins with a precise analytical model for the generation of the synthetic dataset.

The solution of the analytical model is defined by the electrical and magnetic properties of a coin, which allows the flexibility in datasets generation for selectable levels of difficulties in the datasets by varying the estimated electrical and magnetic properties of a coin, thus providing the flexibility in replicating a scenario for the generation of counterfeit coins with different qualities. The use of different levels of difficult datasets in NN also allows investigating the limits of classification accuracy, if the dataset has features too close in regard to the coin properties.

For the elaboration of NN, different frameworks exist [1]. Researchers need to select one of the frameworks suitable for the targeted application [2]. The selection of a suitable framework is an important issue because it decides the level of accuracy, which can be reached, and increases the certainty about the reached classification results. If the researcher uses the unsuitable framework, the results reached could be taken as representative even if they are not the best. In many situations, it remains unclear if the dataset is not sufficient or the neural network structure is not suitable for the dataset, or the training procedure did not work well. Identifying the suitable framework for the targeted application increases the reliability of results, therefore, leading to more certain knowledge. Therefore, it is necessary to estimate the performance of each framework under the same conditions for the targeted application in regard to the different performance metrics such as prediction accuracy, model size, prediction time, and model training time. Based on the performance of a framework, in-regards to the targeted application, one should select a certain framework. For this purpose, a systematic comparison of the frameworks is necessary.

### 1.1. Related Works in Machine-Learning

Until now, most of the qualitative and quantitative comparative studies for different frameworks such as Caffe, TensorFlow, CNTK, Pytorch, Theano, Neon, and MXNet are based on the image, pattern, or natural language processing datasets [1,3,4,5,6,7,8,9,10,11]. Al-Bdour et al. [1] compared three different deep-learning frameworks namely TensorFlow, Theano, and Microsoft’s CNTK for the image and Natural Language Processing (NLP) datasets. The performance of each framework was evaluated on a laptop with Intel Core i7 processor, 16 GB RAM, 64-bit operating system, and x64-based processor. It was observed that the CNTK was performing superiorly for most of the trained datasets. Bahrampour et al. [11] compared five different frameworks ‘Caffe, Neon, TensorFlow, Theano, and Torch’. The comparison was carried out on CPU and GPU by training the convolutional networks on the MNIST and ImageNet datasets. They concluded that the Caffe was the easiest when emphasizing ease of use. In terms of performance, they noticed that Torch was the best for training and testing their DL architectures on a CPU platform while Theano came in second and Neon gave the worst performance. In image processing and pattern recognition, used datasets are present in the form of matrices thus the datasets are present in 2-dimensions [12]. On the contrary, the dataset based on impedance spectroscopy is present in the form of a one-dimensional array, thus having a different format as compared to image datasets. Until now, mostly the comparative study of different frameworks for the training of NN models is implemented using image datasets. Therefore, there is a need for the comparative study of the different frameworks using a sensor dataset based on impedance spectroscopy, for the evaluation and selection of frameworks with better performance in regard to different performance metrics.

### 1.2. Related Works in Eddy Current Sensors

A wide range of applications for eddy current sensors with machine-learning has been implemented, such as proximity sensing [13], force sensing, distance sensing [14], and metal thickness estimation [15]. For instance, in [16], a support vector machine ‘supervised machine-learning algorithm’ has been implemented for classifying bi-metallic coins using an eddy current sensor. Ramos et al. [15] used inductive sensors to determine metal plate thickness together with a support vector machine (SVM) that classified their data into multiple classes of different thickness levels. Kantor et al. [14] showed that ANNs could be used for material independent distance estimation with output errors from 1% to 3% depending on the sensor-to-target distance. Since eddy current sensors are useful in the characterization of metals with different magnetic properties, therefore, in this paper an application for the classification of bi-metallic coins is selected to separate the coins with similar mechanical properties. Due to the limited availability of a sufficient number of coins to generate a testing dataset, an eddy current sensor coil together with an analytical model is used to estimate the properties of coins and thereafter, the novel method based on interpolation technique is used to synthesize the artificial dataset with different levels of difficulty in the dataset for neural network (NN) learning and its performance evaluation.

### 1.3. Methodology

**Figure 1:**
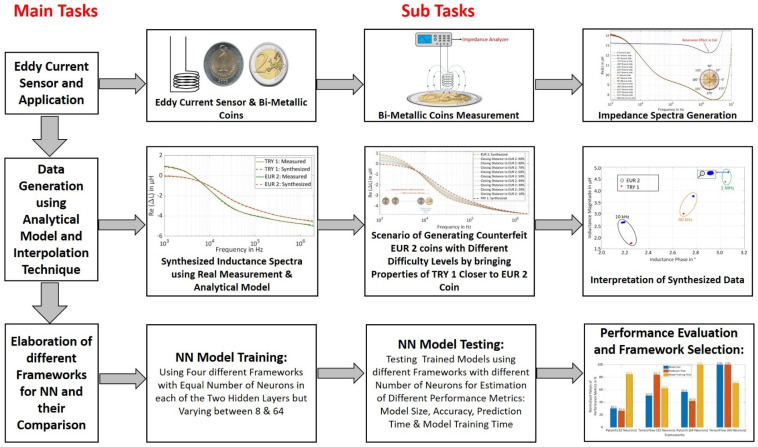
Methodology of Investigations for Four Frameworks ‘Keras with TensorFlow, Pytorch, TensorFlow, and CNTK’.

In this paper, four different machine-learning frameworks ‘Keras with TensorFlow at the backend, Pytorch, TensorFlow, and CNTK’ are selected for quantitative comparison based on different performance metrics for a trained NN model. The dataset for NN training is artificially synthesized (s. Figure 1) for a EUR 2 coin and TRY 1 coin using an eddy current sensor and analytical model together with a novel method based on interpolation technique which, drifts the properties of TRY 1 coin closer to EUR 2 coins, thereby generating a scenario for the different qualities of counterfeit EUR 2 coins.

For NN training, the dataset is generated in Section 2 using an application of an eddy current sensor for the ‘EUR 2 and TRY 1’ coins. The selected coins have nearly the same mechanical and geometrical properties and thus the coin with a lower economic value, i.e., TRY 1 can be used as a counterfeit coin for the coin with higher economical value, i.e., EUR 2 in the vending machines. Thus, there is a need for coin classification with upgraded technology to avoid counterfeiting and misusing of lower economical value coins in place of higher economical values coins [16]. Moreover, bi-metallic coin consists of security features within the layered structure of coins with different properties. These coins comprise two different types of metal with different properties, thus generating a complex scenario for the classification of the coins by extracting different properties of a coin from different layers depending upon the used frequencies for a particular penetration depth of the eddy current effect in the coin. The obtained information from the impedance spectra will be given as input to the NN for using the internal structure and security features of the bi-metallic coins.

Since the counterfeit EUR 2 coins with different qualities are not commonly available and are also illegal to possess counterfeit coins unless provided by Federal Bank, therefore, TRY 1 coin with the most similar properties to that of EUR 2 coin is used for the artificial generation of the dataset. Moreover, we do not have access to the huge number of TRY 1 coin and therefore to keep the balance between the use of EUR 2 and TRY 1 coins, an analytical model is used to synthesize the artificial dataset clusters for learning of neural networks.

After generating the artificial dataset, the difficulty level in the dataset is to be increased to generate a new dataset for model training. Until now, the oversampling technique is widely used where new data points are generated within the dataset for balancing the dataset with equal contribution of data points from all the used classes [17]. However, there is no technique used to generate a new dataset with increased difficulty using the current dataset for model training. Therefore, in this paper, for the generation of a new difficult dataset from existing data points, an interpolation technique is used to drift the properties of both used coins closer to each other. This step aims to bring the magnetic properties and other parameters of coins closer to each other to replicate the scenario with a good imitation of EUR 2 coins and to test the limit of prediction accuracy for all the used frameworks with an increase in the difficulty level of the dataset. In succession, interpretation of generated synthetic dataset is carried out in Section 3, for instance, data visualization at selected frequency points and calculation of feature score for the most contributing feature in the classification. In [1,8,9,10] three frameworks and in [11] five frameworks are compared. By having insight into the results of these papers, frameworks with promising results for different performance metrics are selected for comparison in our work. Frameworks, which will receive continuous development support in the future, are used in our work. The frameworks are also selected keeping in mind the library support for the portability of the trained model to micro-controller in the future. The NN implementation is explained in Section 4, where supervised machine learning is implemented on the aforementioned frameworks using feed-forward NN consisting of two hidden layers and each hidden layer consists of an equal number of neurons varying between 8 and 64. The number of neurons in the hidden layers is varied to understand its effect on different mentioned performance metrics. The evaluation of the frameworks is based on different performance metrics namely prediction time, prediction accuracy, model size, and model training time. Results are discussed in Section 5, explaining the performance of different metrics for different frameworks and the effect of varying neurons in the hidden layer on the performance of compared metrics. In Section 6, we conclude our findings for the selection of a framework with better performance.

## 2. Principle of Eddy Current Sensor and Application in Coin Classification

For the implementation of neural networks, it is recommended to use a huge dataset for model training, to achieve better prediction accuracy. It is difficult to gather such a dataset solely by measurement process and there are no readily available sensor-based datasets for bi-metallic coins, which can be modified to increase the difficulty levels to create a scenario similar to that of counterfeit EUR 2 coins and also for testing the limits of prediction accuracy. Therefore, an approach is used which can artificially synthesize datasets using real measurements of eddy current sensor coil for the available coins and the analytical model.

### 2.1. Eddy Current Measurements on Bi-Metallic Coins

**Figure 2:**
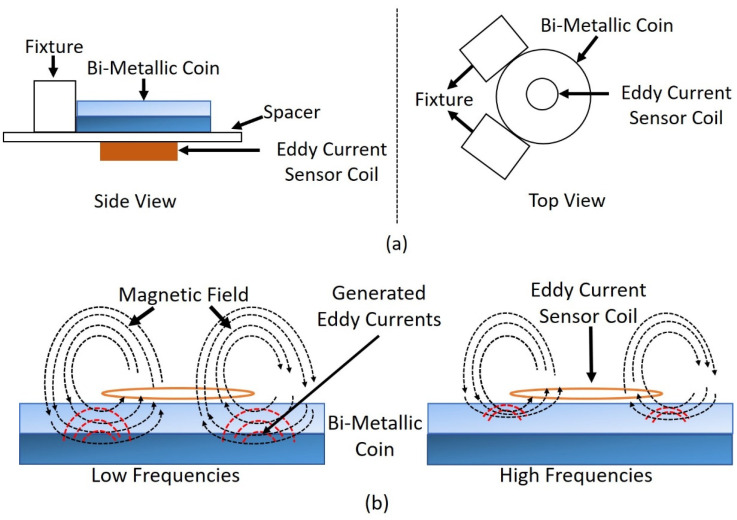
Representation of Eddy Current Sensor Coil over Bi-Metallic Coin (**a**) Schematic of Measurement Set-Up. (**b**) Side View of Magnetic Field Excitation at Different Frequencies.

For this purpose, ‘EUR 2 and TRY 1’ bi-metallic coins are chosen, which consist of two metal layers in the centerpiece with similar geometrical and mechanical properties [16]. The outer ring of a EUR 2 coin is made from copper and nickel while the center layer consists of nickel and brass. Similarly, the outer ring of TRY 1 is made from copper, nickel and zinc and the center layer consists of copper, nickel and zinc but in different proportions as that of the outer ring. A commercial eddy current sensor coil with an inductance value of 13 µH, an outer diameter of 10 mm and a height of 1.6 mm was used for this application. The sensor coil was placed co-axially over the coin so that the center of the coin overlaps with the center of the coil to physically measure the inductance values in the presence of both coins as shown in Figure 2a. The measurement process was carried out using an Impedance Analyzer in the frequency range of 1 kHz to 10 MHz to extract the information from different layers of the coin with varying penetration depths depending upon the frequency [16] as shown in Figure 2b.

**Figure 3:**
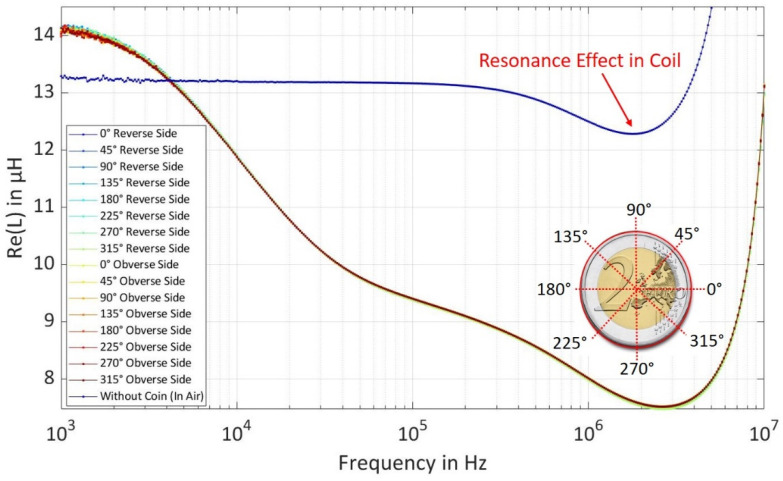
Measured Inductance Spectra of EUR 2 Coin using Reverse and Obverse Side at different Rotating Angles from 0° to 360° Spanned each at 45°.

The coins were measured on both the obverse side and the reverse side at varying angles each spanned at 45° from 0° to 360° to prove the rotation invariance of the coin irrespective of the rotated placement angle over the coil. Figure 3 shows the real part of inductance spectra for EUR 2 coins measured at different angles, it can be seen that the inductance spectra overlap with each other and have similar inductance irrespective of the placement angle of the coin which proves that there is no dependency on the direction of placement of coin above the coil. The different measured inductance spectra of both coins at different angles result in variations of the inductance spectra. The statistics ‘mean and standard deviation’ from the variation of the inductance spectra can be calculated which contributes to the generation of synthetic dataset cluster by extracting the magnetic properties of both bi-metallic coins in different layers using an analytical model, which is explained in a later stage in this section.

### 2.2. Analytical Modelling and Parameter Extraction

The analytical model was implemented in MATLAB, which is based on Dodd and Deeds model [18]. The model calculates the inductance of eddy current coil above two-layer target material. Measured inductance spectra over the coin were given as input to the analytical model and the model interpreted different magnetic properties of coins (conductivity of layer 1 and layer 2, the permeability of layer 2), distance from coil center to end of layer 1 in a coin and thickness/depth of layer 1 in a coin. Using the estimated parameters, magnetic vector potential was calculated by Equation (1) [19] and the inductance of a coil with ‘N’ number of turns was calculated over both coins in the frequency range of 1 kHz to 2 MHz using Equation (4). The frequencies above 2 MHz are neglected due to the presence of resonance effect in the coil as shown in Figure 2.

(1) $$A\left(r,\;z\right)=\frac{\mu_{0}{\mathrm{Ir}}_{0}}{2}\;\overset{\infty }{\underset{0}{\int }}J_{1}\left({\alpha r}_{0}\right)\cdot {\;J}_{1}\left(\alpha r\right)\cdot e^{-\alpha_{0}l-\alpha_{0}z}\cdot \frac{\alpha }{\alpha_{0}}\left\{e^{2\alpha_{0}l}+\left[\frac{\left(\alpha_{0}+{ß}_{1}\right)\left({ß}_{1}-{ß}_{2}\right)+\left(\alpha_{0}-{ß}_{1}\right)\left({ß}_{1}+{ß}_{2}\right)e^{2\alpha_{1}c}}{\left(\alpha_{0}-{ß}_{1}\right)\left({ß}_{1}-{ß}_{2}\right)+\left(\alpha_{0}+{ß}_{1}\right)\left({ß}_{1}+{ß}_{2}\right)e^{2\alpha_{1}c}\;}\right]\right\}d\alpha$$

(2) $$\alpha_{i}=\sqrt{\alpha^{2}-\omega^{2}\mu_{i}\in_{i}+j\omega \mu_{i}\sigma_{i}}$$

(3) $$\beta_{i}=\frac{\mu_{0}}{\mu_{i}}\cdot \alpha_{i}$$

(4) $$L=\frac{A\cdot 2\cdot \pi \cdot r\cdot N^{2}}{I}$$

where: A: Magnetic vector potential, r: Radius of wire surface, z: Height of a coil above the coin surface, *µ*_0_: Permeability of air, I: Excitation current, r_0_: Average radius of the coil, J_1_: First order Bessel function, α: Separation constant, l: Distance of the center of the coil to the coin surface, c: Thickness of the top layer of the coin, *ω*: Angular frequency, *i*: integer ‘0-2’, *µ*_1_: Permeability of the top layer of the coin, *µ*_2_: Permeability of the center layer of the coin, *∈*_0_: Permittivity of the air, *∈*_1_: Permittivity of the top layer of the coin, *∈*_2_: Permittivity of the center layer of the coin, *σ*_0_: Conductivity of the air, *σ*_1_: Conductivity of the top layer of the coin, *σ*_2_: Conductivity of the center layer of the coin, L: Inductance of the sensor coil, N: Number of turns of the coil

**Figure 4:**
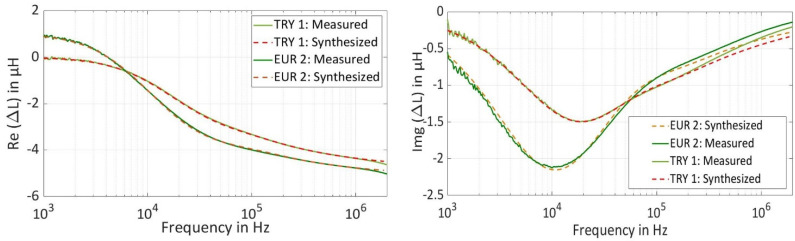
(**a**) Real Value of Measured and Synthesized Intermediate Inductance Spectra for EUR 2 and TRY 1 Coin. (**b**) Imaginary Value of Measured and Synthesized Intermediate Inductance Spectra for EUR 2 and TRY 1 Coin.

In Figure 4, spectra for the real part and the imaginary part of intermediate inductance (ΔL) are plotted using the measured spectra and synthesized spectra, which are generated by the analytical model. Intermediate inductance is calculated using Equation (5), by subtracting the estimated inductance of a coil-over a coin (∆L_Coin_) from inner inductance inside the wire (∆L_Air_) due to skin effect, which is a coil in the air without target material [20,21]. The measured spectra and synthesized spectra using the analytical model are identical and fit to each other over the entire frequency range.

(5) $$\Delta L={\;L}_{\mathrm{Coin}}-{\;L}_{\mathrm{Air}}$$

The selection of frequency is important to understand different magnetic property information from each layer of the coin. The three frequency points ‘10 kHz, 40 kHz, 1.1 MHz’ are selected based on the sensitivity analysis, which provides enough information required to successfully classify the coins in accordance to the penetration depth of the magnetic field to access the layered structure of the coins [16]. These three frequencies result in a pair of three real and imaginary values, which are converted to inductance amplitude-phase pairs using Equations (6) and (7), resulting in a feature set of six.

(6) ΔLAmp=ΔLRe2+ ΔLImg2

(7) ΔLPhase=tan−1(ΔLImgΔLRe)

The process of parameter estimation was repeated for both the coins using different measured inductance spectra at different angles. Since the coins were measured at 8 different angles on each side, therefore 16 inductance spectra were measured for each coin and thus each parameter of the coin was estimated 16 times. Using these values, the mean and standard deviation of each parameter was calculated.

In the next step, the cluster of intermediate inductances was generated at the selected frequency points for both coins. The mean values and standard deviations of predicted parameters were used in the random function of Matlab with normal distribution to generate 10,000-inductance amplitude-phase pairs at one particular frequency for each coin.

### 2.3. Interpolation Technique to Generate Datasets with Different Challenging Levels

**Figure 5:**
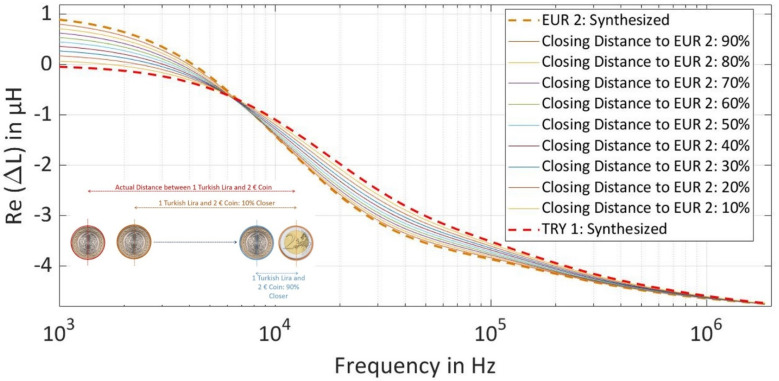
Real Values of Synthesized Intermediate Inductance Spectra at different Difficulty Levels of TRY 1 Coin by Bringing the Parameters of Coin Closer to EUR 2 Coin.

After generating the inductance amplitude-phase values at all the three selected frequencies, the difficulty level in the dataset was increased by interpolating the estimated parameters between the parameter values of TRY 1 and EUR 2 coins to make the prediction task more difficult for the NN. The purpose of increasing the difficulty level is to understand the limits of the prediction accuracy by bringing both coins closer to each other in regards to the magnetic properties, which can also replace one coin as a counterfeit coin for the other as can be seen in Figure 5.

For the generation of increased difficulty level datasets, the mean value of all the estimated parameters ‘conductivity of layer 1 and layer 2, the permeability of layer 2, distance from coil center to end of layer 1 in a coin, and thickness/depth of layer 1 in a coin’ for both coins was used. In the first step, the absolute distance between each parameter of both the coins was calculated using Equation (8) and then the unit vector was calculated using Equation (9).

(8) $$\left|{\mathrm{Distance}}_{\mathrm{Parameter}}\right|=\sqrt{{\left({\mathrm{Mean}\;\mathrm{Value}}_{\mathrm{Parameter}\_\mathrm{Eur}\;2}-{\;\mathrm{Mean}\;\mathrm{Value}}_{\mathrm{Parameter}\_\mathrm{TRY}\;1}\right)}^{2}}$$

(9) $$\mathrm{Unit}\;\mathrm{Vector}=\frac{\left|{\mathrm{Distance}}_{\mathrm{Parameter}}\right|}{\left({\mathrm{Mean}\;\mathrm{Value}}_{\mathrm{Parameter}\_\mathrm{Eur}\;2}-{\;\mathrm{Mean}\;\mathrm{Value}}_{\mathrm{Parameter}\_\mathrm{TRY}\;1}\right)}$$

In the next step, the mean value of all the estimated properties of TRY 1 is shifted in the direction of EUR 2 coins by using the formula in Equation (10). The closing distance of TRY 1 to EUR 2 depends on the scaling factor ‘k’, where k = 90% is closest to EUR 2 coin.

(10) $${\mathrm{New}\;\mathrm{Mean}\;\mathrm{Value}}_{\mathrm{Parameter}}={\mathrm{Mean}\;\mathrm{Value}}_{\mathrm{Parameter}}+\mathrm{Unit}\;\mathrm{Vector}\;\cdot \left|{\mathrm{Distance}}_{\mathrm{Parameter}}\right|\cdot \;k$$

After finding the new position of estimated parameters of TRY 1 in the vector plane, the standard deviation was applied similarly as before to generate a cluster of new inductance amplitude-phase pairs each with 10,000 points at all three frequencies for all closing distances.

## 3. Interpretation of Synthesized Data

**Figure 6:**
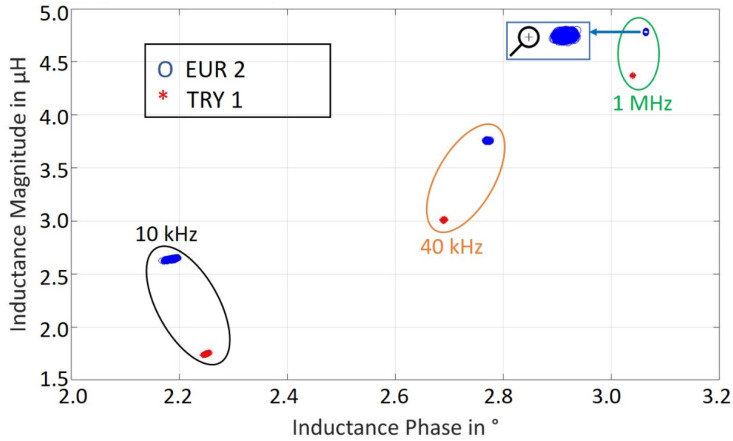
Inductance Ratio vs. Inductance Phase for EUR 2 and TRY 1 Coin Classes at Three Frequencies Measured 10,000 Times Each.

The segregation of coins into different classes can be difficult using only inductance amplitude or inductance phase at different frequencies [16]. For this reason, the pair of inductance amplitude-phase at three selected frequencies are to be used for the classification as shown in Figure 6, which leads to six features per coin class. There is a total of 10,000 points for each coin inductance amplitude and phase at a single frequency. Since three frequency points are used, therefore synthesized dataset consists of 60,000 points for each coin class. The total dataset for both coins have 120,000 data points.

It is a common practice in machine learning to normalize the given raw data before using it for training to mitigate the effect of one feature with more variation on the prediction results [16]. For this purpose, the standard-scaler function in Equation (11) is used. It shifts the distribution of raw data to have a mean of zero and a standard deviation of one without changing the type of data distribution.

(11) $$z=\frac{x-\mu }{s}$$

where z is the normalized value of a feature, x is the raw value of a feature, µ is the mean value of the feature and s is the standard deviation of the feature.

To devise features that lead to good separating hyperplanes and find the most contributing features in the classification, a statistical method χ^2^ (chi^2^) is used [22]. The calculated scores for all normalized six features show that the phase at 10 kHz and 40 kHz contributes the most in classification as can be seen in Table 1.

## 4. Implementation and Training of Neural Network Frameworks

This paper aims to compare four different machine learning frameworks ‘Keras with TensorFlow at the backend, Pytorch, TensorFlow, and CNTK’ using feed forward NN for an artificially synthesized dataset using eddy current sensor. Keras is a high-level application programming interface (API) and can be used on top of low-level API such as TensorFlow and CNTK. In this work, the framework Keras is used with TensorFlow at the backend thus operating as high-level API while all other used frameworks are low-level API.

High-level API are designed for quick prototyping and provide a convenient, higher level of abstraction where a user can focus on developing and training neural networks rather than dealing with low-level operations. However, with too many levels of abstraction, debugging the NN often proves to be difficult. On contrary, low-level API’s have a lower level of abstraction resulting in a complicated programming structure with less readability but also providing the user with more degree of freedom in the case of debugging. Different available low-level API frameworks have a different methodology for network execution. For instance, some low-level API frameworks support data and model parallelism, and others do not, some low-level API’s support static computational graphs, which means one cannot change the parameter of the NN on go while some supports dynamic computational graph where execution is performed as operations are defined thus network can process variable-length inputs and outputs. For model training/learning, Python, a programming language, was used for all the frameworks. Initially, the complete dataset was shuffled and 80% of the dataset was separated from a total dataset in a way to achieve a balanced dataset thus containing an equal number of data points from each class. Ten percent of the training dataset was reserved for the model validation.

The learning process of neural networks is viewed as the problem of updating network architecture and connection weights from the training data. The performance of the NN is improved over time by iteratively updating the weights in the network. The output of the NN can be adjusted according to the desired output by modifying the connection weights and thus reducing the error. For this purpose, a back-propagation learning algorithm is used, which updates the weights from a small random value to a certain value where the error in the output layer reaches below a specified threshold value.

Different multiple combinations of hyper-parameters were evaluated to select one combination of hyper-parameters, which achieves comparable training accuracy for all the selected frameworks [1,23]. For a network to learn complex data, ‘rectified linear unit’ activation function is used, which is better performing activation function as compared to other activation functions [24].

**Figure 7:**
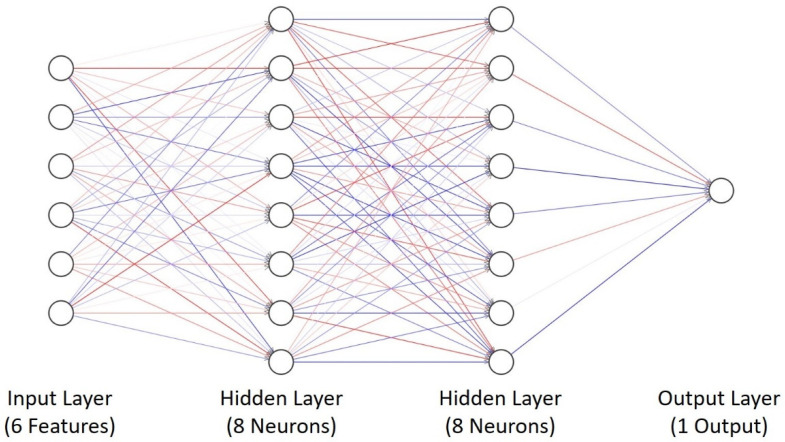
Structure of NN Implemented for 6 Features and 1 Output with 2 Hidden Layers Each Containing 8 Number of Neurons.

An implemented NN consists of two hidden layers as shown in Figure 7 with equal number of neurons in both layers but a varying number of neurons between 8 and 64 to understand its effect on different performance metrics. However, it is possible to use a single hidden layer, which is sufficient to represent any function. The term is coined as a universal approximation theorem, but the layer may be infeasibly large, may fail to learn and generalize correctly [25], and can also be prone to overfitting. It is proven that the NN with two hidden layers performs better as compared to a network with one hidden layer only [26] and can better generalize the unseen data as well.

The number of epochs for model training was fixed at 10 in all the frameworks with an exception of 100 epochs in CNTK to achieve similar prediction accuracy as of other frameworks. Two dropout layers with a dropout ratio of 0.3 were used to avoid overfitting. Adaptive moment estimation (Adam) optimizer was used for tuning the weights and minimizing the sparse categorical cross-entropy loss. The output layer in all frameworks uses ‘sigmoid’ as an activation function. The used hyper-parameters of the implemented neural network can be seen in Table 2. 

The aforementioned frameworks were evaluated on a laptop with Windows 10 operating system. The laptop has a quad-core Intel i7-6700HQ processor with a hyper-threading clock of 2.60 GHz and 16 GB RAM.

The performance of each framework is evaluated by the metrics: model size, model training time, prediction time, and prediction accuracy.

## 5. Results

In this section, the performances of the trained feed-forward neural network models on different frameworks are evaluated for the synthesized dataset from Section 2. For performance analysis, the number of neurons in two hidden layers was always kept equal and varied between 8 and 64. The model accuracy is predicted by varying the number of neurons on the different difficult synthesized datasets by bringing the magnetic properties of TRY 1 coin near to those of the EUR 2 coins. Model size, prediction time, and model training time were measured by varying the number of neurons for the actual distance between TRY 1 and EUR 2 coins as a dataset with increased difficulty level yields the same results and does not depend on the difficulty level of the dataset.

### 5.1. Model Size

**Figure 8:**
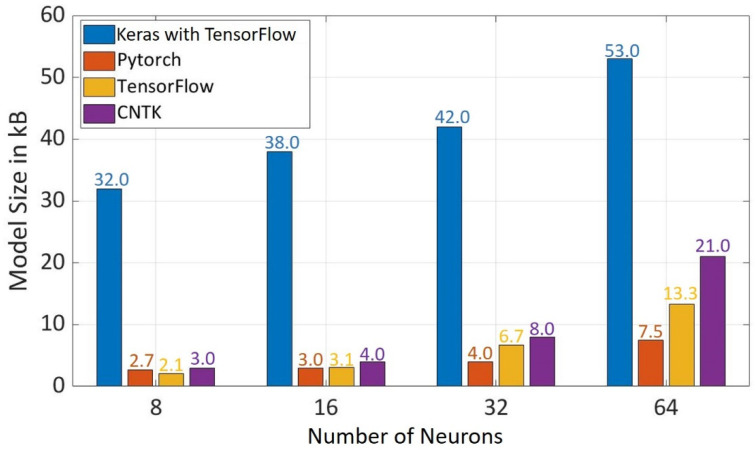
Model Size for Different Frameworks using Different Number of Neurons in Hidden Layers.

It was observed in Figure 8 that the model size increases with the increase in the number of neurons in hidden layers. Keras with TensorFlow at the backend has the biggest model size at all the evaluated number of neurons, which is due to the reason that it is a high-level API. In the case of low-level API, Pytorch has the smallest model size of 3 kB for 16 neurons, 4 kB for 32 neurons, and 7.5 kB for 64 neurons. There is an exception for 8 neurons where TensorFlow has the smallest model size of 2 kB. In regard to the model size, Pytorch has better performance than other frameworks.

### 5.2. Model Training Time

**Figure 9:**
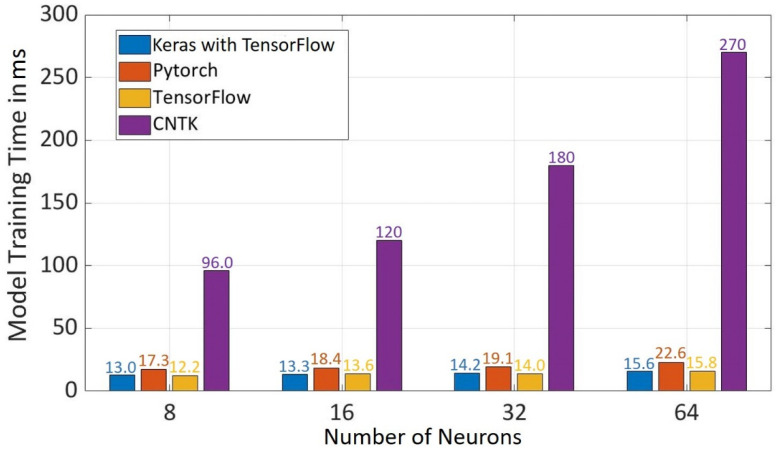
Model Training Time for Different Frameworks using Different Number of Neurons in Hidden Layers.

The model training time increases with the increase in the number of neurons in hidden layers (s. Figure 9). TensorFlow has the least training time followed by Keras with TensorFlow while CNTK has the highest training time of all frameworks for all neurons in the hidden layer, which is due to the reason that 100 epochs are used for CNTK model training.

### 5.3. Prediction Time

**Figure 10:**
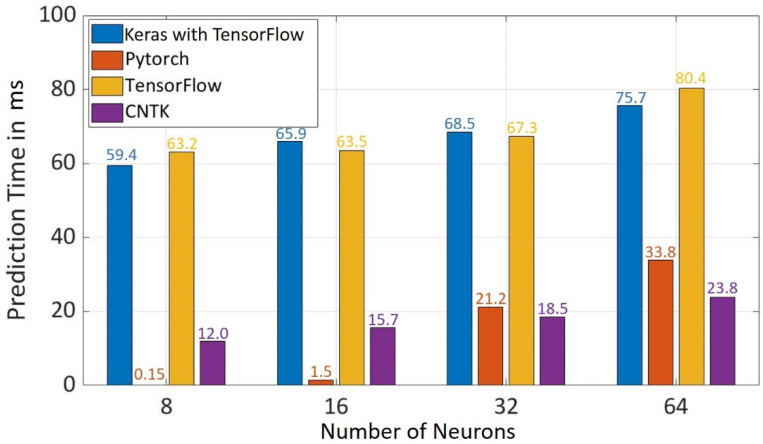
Prediction Time for Different Frameworks using Different Number of Neurons in Hidden Layers.

Prediction time is the time taken by the model to predict the class of tested data. It was observed in Figure 10 that as the number of neurons increases, prediction time increases. Pytorch and CNTK have the shortest prediction time depending upon the neurons in the hidden layer. Pytorch has the shortest prediction time of 0.15 ms for 8 neurons and 1.5 ms for 16 neurons while CNTK has the shortest prediction time of 18.5 ms at 32 neurons and 23.8 ms at 64 neurons. Similarly, TensorFlow and Keras with TensorFlow have the longest prediction time depending upon neurons in the hidden layer. TensorFlow has the longest prediction time of 63.2 ms with 8 neurons and 80.4 ms with 64 neurons while Keras with TensorFlow has the longest prediction time of 65.9 ms with 16 neurons and 68.5 ms with 32 neurons. Therefore, in regards to the prediction time, Pytorch has a better performance for the model with 8 and 16 neurons while CNTK has better performance for the model with 32 and 64 neurons.

### 5.4. Prediction Accuracy

**Figure 11:**
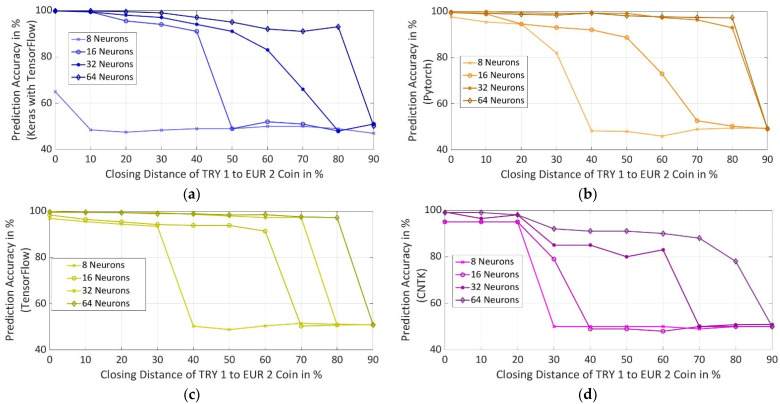
Prediction Accuracy at Different Closing Distances between TRY 1 and EUR 2 Coins using Different Number of Neurons in Hidden Layers for Different Frameworks: (**a**) Keras with TensorFlow Backend (**b**) Pytorch (**c**) TensorFlow (**d**) CNTK.

Prediction accuracy was calculated for different difficult synthesized datasets for different numbers of neurons in hidden layers of a particular framework. The difficulty of the dataset was increased to understand which framework outputs the best accuracy, if both the coins have nearly the same magnetic properties and other parameters, as mentioned in Section 2. It was observed in Figure 11, as the closing distance between the coins is increased, the accuracy of all the frameworks for all used number of neurons reduces after a certain closing distance as a machine cannot further create accurate decision boundaries after a certain level. It was also observed that for all the frameworks, prediction accuracy increases with the increase in the number of neurons. For this purpose, the prediction accuracy of each framework is discussed further in regard to the number of neurons in the hidden layers.

#### 5.4.1. NN with 8 Neurons in Each Hidden Layer

Keras with TensorFlow backend has the least accuracy out of all the frameworks followed by CNTK. Pytorch has the highest accuracy of 97.66% followed by TensorFlow at 96.83% for actual synthesized properties of the coins. As the closing distance between the coins increases, accuracy starts to drop. For both Pytorch and TensorFLow, accuracy drops to around 50% when coins are 40% or closer to each other in terms of magnetic properties. There is no further drop in the accuracy above 40% closing distance and was restricted to around 50%.

#### 5.4.2. NN with 16 Neurons in Each Hidden Layer

For actual synthesized properties of the coins, Keras with TensorFlow has the highest accuracy of 99.8%, followed by 99.51% in Pytorch, 98.33% in TensorFlow, and 95% in CNTK. As the closing distance is increased from 10% to 40%, the frameworks have accuracy above 91% except for CNTK for which accuracy drops to 49%. For Keras with TensorFlow, the accuracy drops to around 50% at the closing distance of 50%. Pytorch and TensorFlow see the drop in accuracy to around 50% from the closing distance of 70%. At 50% and 60% of the closing distance between the coins, TensorFlow has better accuracy in comparison to Pytorch.

#### 5.4.3. NN with 32 Neurons in Each Hidden Layer

Pytorch achieves the highest accuracy of 99.93% followed by TensorFlow at 99.86%, Keras with TensorFlow backend at 99.8% and CNTK at 99.23% for actual synthesized properties of the coins. Pytorch attains the highest accuracy of 92.93% in comparison to other frameworks as the closing distance between the coins is increased to 80%. TensorFlow also shows good prediction accuracy up to 97.31% with the increasing closing distance until 70%. Keras with TensorFlow has decreasing accuracy with an increase in closing distance between the coins and accuracy falls below 90% at a closing distance of 60% and reaches below 50% at a closing distance of 80%. CNTK achieves an accuracy of 98% up to 20% closing distance, from the closing distance of 30% to 60%, accuracy is between 80% and 90%, and from 70% to 90% of the closing distance, accuracy falls below 50%.

#### 5.4.4. NN with 64 Neurons in Each Hidden Layer

Keras with TensorFlow achieves an accuracy in the range of 99.8% to 93% up to the closing distance of 80% in between the coins and drops to 50% for a closing distance of 90%. The accuracy for CNTK varies from 99% to 78% for a closing distance up to 80% and drops to 50% at a closing distance of 90%. Pytorch and TensorFlow have almost similar performance in accuracy for all the closing distances. The accuracy for both frameworks varies from 99.6% to 97.2% for a closing distance up to 80% and decreases to 50% for a closing distance of 90%.

### 5.5. Framework Evaluation

After comparing all the frameworks for different performance metrics, it was observed that the Keras with TensorFlow and CNTK have the worst performance for most of the compared metrics. Pytorch and TensorFlow with 8 and 16 neurons do not yield good prediction accuracy. Therefore, the major comparison is restricted to Pytorch and TensorFlow for 32 and 64 neurons in the hidden layers.

**Figure 12:**
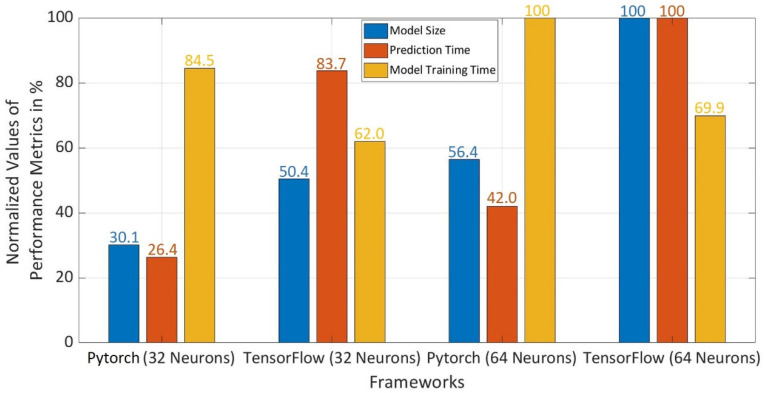
Comparison of the Performance Metrics considering Model Size, Prediction Time and Model Training Time for Pytorch and TensorFlow with 32 and 64 Neurons in each Hidden Layers.

For this purpose, the first criteria for the selection of a better-performing framework are considered to be prediction accuracy. It was observed in Figure 11b,c that Pytorch with 32 neurons has a prediction accuracy, which is lying in the same range as that of Pytorch and TensorFlow with 64 neurons. After comparing the accuracy, three performance metrics namely model size, prediction time, and model training time were normalized to select a better-performing framework as shown in Figure 12. Pytorch with 32 neurons has only a 16.5% reduction in model training time as compared to Pytorch with 64 neurons. However, model training time becomes insignificant for the selection as Pytorch with 32 neurons has a reduction in model size by 70% and 16.3% as compared to TensorFlow and Pytorch with 64 neurons. It is also 73.6% and 15.6% faster in prediction in comparison to TensorFlow and Pytorch with 64 neurons. Thus, Pytorch with 32 neurons has the smallest model size and shortest prediction time with desired prediction accuracy above 90% in comparison to the Pytorch and Tensorflow with 32 and 64 neurons.

### 5.6. Reliability Evaluation of Selected Framework

**Figure 13:**
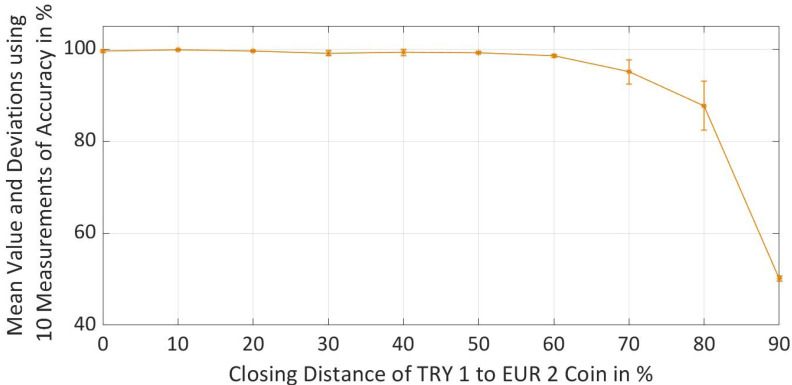
Reliability Evaluation of Pytorch with 32 Neurons in each Hidden Layer by Calculating the Mean Value, Minimum and Maximum Deviation using 10 Measurements for Accuracy Metric.

Pytorch with 32 neurons in each hidden layer was selected as a better-performing framework after the comparison and evaluation based on the different performance metrics. To understand the reliability of a trained neural network for the selected Pytorch framework, the prediction accuracy metric was measured every ten times for all datasets with different difficulty levels. Mean values and standard deviations were calculated for all the datasets and thereby maximum and minimum deviation of the accuracy was calculated as shown in Figure 13. It was observed that the standard deviation increases with the increase in difficulty level of a dataset, especially, when properties of TRY 1 coin are 70% and 80% closer to the EUR 2 coins, while for other distances the standard deviations were lower. At a distance of 90% standard deviation is again improved due to the reason that model prediction has reached saturation and is unable to create accurate decision boundary, thus cannot differentiate properly between the classes and gives the accuracy of around 50%.

## 6. Conclusions

This contribution aims to carry out a comparative study of different frameworks ‘Keras with TensorFlow at the backend, Pytorch, TensorFlow, and CNTK’ for a feed-forward neural network model based on the sensor dataset with different difficulty levels of the bi-metallic coins. Since the sensor dataset based on bi-metallic coins for model training is not readily available, therefore, for this purpose, the application of eddy current sensor was used to artificially synthesize the dataset, which implies the use of two bi-metallic coins ‘EUR 2 and TRY 1’ with similar physical and mechanical properties. The synthesized dataset was generated using measured inductance spectra of coins and an analytical model based on Dodd and Deeds model.

After synthesizing the dataset for both the coins, the level of difficulty was increased in the dataset by bringing the magnetic properties of both coins closer, creating a scenario with a good imitation of EUR 2 coins and understanding the limitations of prediction accuracy by all the frameworks. With the use of a synthesized dataset, the feed-forward neural network model was trained for all the selected frameworks. All frameworks consist of two hidden layers with an equal number of neurons in both hidden layers. The neurons in the hidden layers were varied between 8 and 64 to understand its impact on different performance metrics namely model size, prediction time, model training time, and prediction accuracy. Finally, a more suitable framework regarding overall better performance with the recommended number of neurons in the hidden layers is to be selected.

It was observed that the compared frameworks have a high accuracy performance for a lower level of difficulty in the dataset. As the difficulty in the dataset is raised, there was a drop in the accuracy of CNTK and Keras with TensorFlow depending upon the number of neurons in the hidden layers. It was observed that CNTK has the overall worst accuracy performance with an increase in the difficulty level of the datasets. On the contrary, to the study carried out in [1], where CNTK was a better performing framework, when predictions were carried out on image datasets. Pytorch and TensorFlow with 8 and 16 neurons also do not show promising accuracy performance. Therefore, the main comparison was confined to Pytorch and TensorFlow with 32 and 64 neurons in the hidden layers, where accuracy is higher for all the levels of difficulties in the dataset. The predicted accuracy in the case of Pytorch with 32 neurons for all the datasets where TRY 1 is moving closer to EUR 2 coin in-regards to the electrical and magnetic properties is almost similar to the accuracy in the case of Pytorch with 64 neurons and TensorFlow with 32 and 64 neurons.

It was observed for Pytorch and TensorFlow with 32 and 64 neurons in hidden layers that there is a minor drop in the accuracy with an increase in the difficulty level of the dataset. However, accuracy was above 90% until both the coins were 80% closer to each other in terms of electrical and magnetic properties. In regard to model training time, Pytorch with 32 neurons consumes more time as compared to TensorFlow with 32 and 64 neurons and 16.5% less than Pytorch with 64 neurons. However, model training time becomes insignificant for the selection as Pytorch with 32 neurons shows far better performance for model size and prediction time. The model size was reduced by 70% and 16.3% as compared to TensorFlow and Pytorch with 64 neurons. Pytorch with 32 neurons predicts the class, 73.6% and 15.6% faster in comparison to TensorFlow and Pytorch with 64 neurons. Thus, it can be concluded that the Pytorch with 32 neurons has overall better performance with the lowest memory footprint (model size) and consumes the least time to predict a coin class with the desired accuracy rate above 90%. After the selection of Pytorch with 32 neurons in each hidden layer, reliability analysis was carried out using accuracy performance metric. The accuracy was measured ten times for each difficulty level of datasets. The mean values were calculated along with the standard deviations, which shows very low deviations from the mean value. Therefore, it can be concluded that the selected Pytorch framework with 32 neurons in each hidden layer of NN reacted as a robust network.

Accuracy is depending upon the number of neurons and the actual level of difficulty in the dataset. It was observed that TensorFlow and Pytorch perform generally well with dataset of a higher level of difficulty. However, for specific lower difficulty levels in the dataset, they are outperformed by Keras with TensorFlow depending upon the number of neurons in the hidden layer. This is the possible explanation for the contradicting results in the existing comparative study based on image processing datasets.

## Figures and Tables

**Table 1:** Features Score Calculated using χ2 Test (Higher the score is more contribution of a feature during classification).

Frequency	Score	
	Impedance	Phase
10 kHz	0.00146	6.346198
40 kHz	0.000571	12.16268
1 MHz	0.000091	0.865512

**Table 2:** Applied Hyper-Parameters for NN Model Training.

Hidden Layers	Neurons in Each Layer	Activation Function	Dropout Layers	Dropout Ratio	Activation Function (Output Layer)	No. of Epochs	Optimizer
2	8–64	ReLU (Rectified Linear Unit)	2	0.3	Sigmoid	10, *100 in CNTK	Adam
